# Measurement of Lateral Transmission of Force in the Extensor Digitorum Longus Muscle of Young and Old Mice

**DOI:** 10.3390/ijms222212356

**Published:** 2021-11-16

**Authors:** Keitaro Minato, Yuki Yoshimoto, Tamaki Kurosawa, Kei Watanabe, Hiroyuki Kawashima, Madoka Ikemoto-Uezumi, Akiyoshi Uezumi

**Affiliations:** 1 Muscle Aging and Regenerative Medicine, Tokyo Metropolitan Institute of Gerontology, 35-2 Sakae-cho, Itabashi, Tokyo 173-0015, Japan; kinbensyonen@yahoo.co.jp (K.M.); yoshimt@tmig.or.jp (Y.Y.); tamaki-k@g.ecc.u-tokyo.ac.jp (T.K.); muezumi@tmig.or.jp (M.I.-U.); 2 Department of Regenerative and Transplant Medicine, Division of Orthopedic Surgery, Graduate School of Medical and Dental Sciences, Niigata University, 1-757 Asahimachi-Dori, Tyuo-Ku, Niigata 951-8510, Japan; keiwatanabe_39jp@live.jp (K.W.); inskawa@med.niigata-u.ac.jp (H.K.); 3 Laboratory of Veterinary Pharmacology, Department of Veterinary Medical Sciences, Graduate School of Agriculture and Life Sciences, Tokyo University, 1-1-1 Yayoi, Bunkyo-ku, Tokyo 113-8657, Japan

**Keywords:** skeletal muscle, lateral force transmission, sarcopenia

## Abstract

The main function of skeletal muscles is to generate force. The force developed by myofiber contraction is transmitted to the tendon. There are two pathways of force transmission from myofibers to tendons: longitudinal transmission that depends on tension elicited via the myotendinous junction and lateral transmission that depends on shear elicited via the interface between the myofiber surface and surrounding connective tissue. Experiments using animal muscle and mathematical models indicated that lateral transmission is the dominant pathway in muscle force transmission. Studies using rat muscle showed that the efficiency of lateral force transmission declines with age. Here, the lateral transmission of force was measured using the extensor digitorum longus muscle from young and old mice. Dependence on longitudinal transmission increased in the old muscle, and there was a trend for lower efficiency of lateral force transmission in the old muscle compared to the young muscle. There was a noticeable increase in the connective tissue volume in the old muscle; however, there was no significant change in the expression of dystrophin, a critical molecule for the link between the myofiber cytoskeleton and extracellular matrix. This study demonstrates the measurement of lateral force transmission in mouse muscles and that alteration in force transmission property may underlie age-related muscle weakness.

## 1. Introduction

The primary function of skeletal muscles is to generate force by contraction. Force is originally produced by actin–myosin crossbridge cycling within myofibers. The force exerted by the myofiber is transmitted to the tendon to pull the bone. Force generation and transmission are essential properties of skeletal muscles and are required for exercise, physical activity, and postural maintenance. Two pathways are involved in force transmission from myofibers to tendons [1]. A widely accepted pathway is that force developed in myofibers is transmitted directly to the tendon by tension elicited via the myotendinous junction (MTJ). This pathway is termed longitudinal force transmission. Another pathway is termed lateral force transmission, where the myofiber-derived force is first transmitted to the adjacent connective tissue by shear elicited via the interface between the myofiber surface and connective tissue, and eventually to the tendon. Longitudinal force transmission via the MTJ is thought to be the main pathway of force transmission. However, many muscles across species have myofibers that terminate within the fascicles without reaching the tendon at least at one end. These muscles are called non-spanning myofibered muscles [1]. In contrast, muscles consisting of myofibers that are attached to the tendon via the MTJ at both ends are called spanning myofibered muscles [1]. Because non-spanning myofibers lack MTJs, they should transmit force through lateral force transmission. Using a mathematical model, Sharafi and Blemker investigated which pathway (longitudinal or lateral) dominates in the force transmission of non-spanning myofibers and found that nearly all forces are transmitted through lateral force transmission [2]. Studies have attempted to measure lateral force transmission in living muscles. Using a special “yoke” apparatus, Ramaswamy et al. measured lateral force transmission of the rat extensor digitorum longus (EDL) muscle or mouse anterior tibialis muscle [3]. Zhang and Gao measured the lateral force transmission of the rat EDL muscle by means of a series of tenotomy and myotomy [4]. In both studies, approximately 80% of the force developed by myofibers was transmitted through lateral force transmission, even though the muscles used in the measurement were spanning myofibered muscles [3,4]. Therefore, lateral force transmission was the major pathway for force transmission in the skeletal muscle.

Skeletal muscle mass and strength decline with age, leading to a condition termed sarcopenia. The loss of muscle strength occurs more rapidly than the loss of muscle mass due to aging [5] and low muscle strength is strongly associated with adverse outcomes, such as long hospital stays, increased functional limitations, poor health-related quality of life, and death [6]. Therefore, muscle strength is a more important indicator than muscle mass. Although age-related changes in myofibers have been extensively studied, the mechanisms of age-related decrease in muscle strength remains largely elusive. Importantly, several studies demonstrated no difference in in vitro single-myofiber contractile properties between young and old mice or humans [7,8,9], strongly suggesting that intrinsic myofiber function is preserved during aging. Thus, factors other than myofibers appear to be responsible for the age-related loss of muscle strength. Since changes in the connective tissue (fibrosis and fat infiltration) are a hallmark of old muscle and the force is transmitted laterally through the connective tissue, alteration in lateral force transmission is thought to be involved in age-related muscle weakness. Indeed, lateral force transmission is impaired in old rat EDL muscles [4]. Although rat muscles are relatively large and amenable to a series of tenotomy and myotomy, measuring lateral force transmission in mice would be meaningful because many genetically modified models are available in mice.

In this study, a series of tenotomies and myotomies was performed using EDL muscles from young and old mice to measure lateral force transmission and examine how aging affects it. There was a trend for decreased efficiency of lateral force transmission in old muscles compared to that in young muscles. Thus, the measurement of lateral force transmission in mice is possible and may be useful for investigating possible mechanisms of muscle weakness in mouse models.

## 2. Results

**Figure 1:**
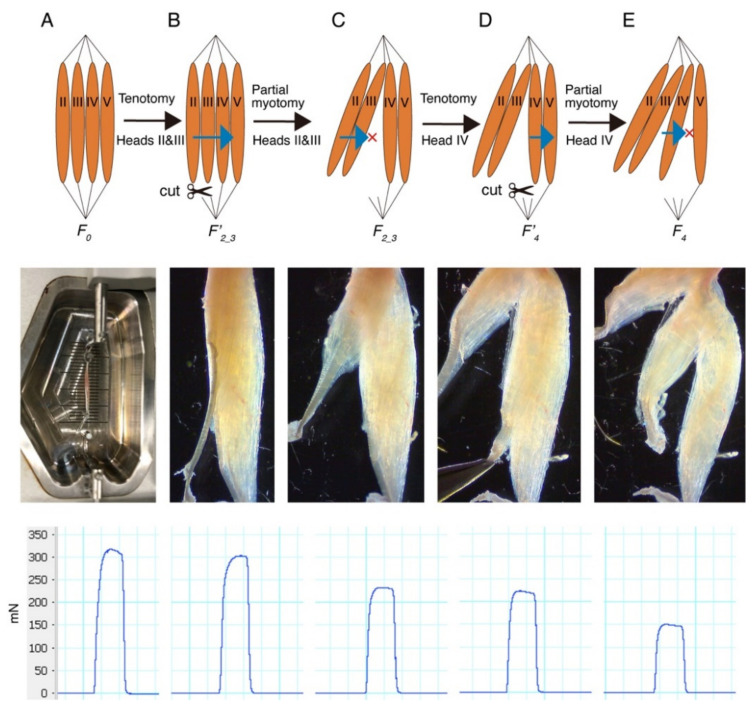
The principle and method for the measurement of lateral force transmission using the mouse extensor digitorum longus (EDL) muscle. (**A**) The maximum tetanic force of the whole EDL muscle was measured and recorded as *F_0_*. (**B**) Force measured after the tenotomy of heads II and III was recorded as *F′_2_3_*. (**C**) Force measured after the myotomy of heads II and III was recorded as *F_2_3_*. (**D**) Force measured after the tenotomy of head IV was recorded as *F′_4_*. (**E**) Force measured after the myotomy of head IV was recorded as *F_4_*. The top row is schematic illustration, the middle row shows the actual setup, and the bottom row shows typical force tracing. The blue arrows in the top row represent lateral force transmission from muscle head(s) with tenotomy to adjacent muscle head(s) having intact tendon(s). The red crosses represent interrupted force transmission by myotomy.

The EDL muscle consists of four heads with distal insertions on digits II–V of the foot. Lateral force transmission can be examined by measuring the maximum tetanic force in each step during a series of tenotomy and myotomy of the EDL muscle [4]. The principle and method of the measurement is illustrated in Figure 1. Since the mouse EDL muscle is small, it is difficult to separate head II from head III without damaging the muscle. Therefore, heads II and III were treated as one head without separating them. First, the maximum tetanic force of the whole EDL muscle was measured and recorded as *F_0_* (Figure 1A). Subsequently, the tendons of heads II and III were cut, and the same electrical stimulation was applied to the whole muscle. This tenotomy procedure could eliminate the longitudinal transmission of force through the tendons of heads II and III. The measured force was recorded as *F′_2_3_* (Figure 1B). Next, heads II and III were separated from head IV, and the same electrical stimulation was applied again. The force measured after this myotomy procedure was recorded as *F_2_3_*, which was the force after the elimination of both longitudinal and lateral force transmission from heads II and III (Figure 1C). Similar tenotomy and myotomy procedures were applied to head IV, and the force measured after each step was recorded as *F′_4_* and *F_4_*, respectively (Figure 1D,E).

**Figure 2:**
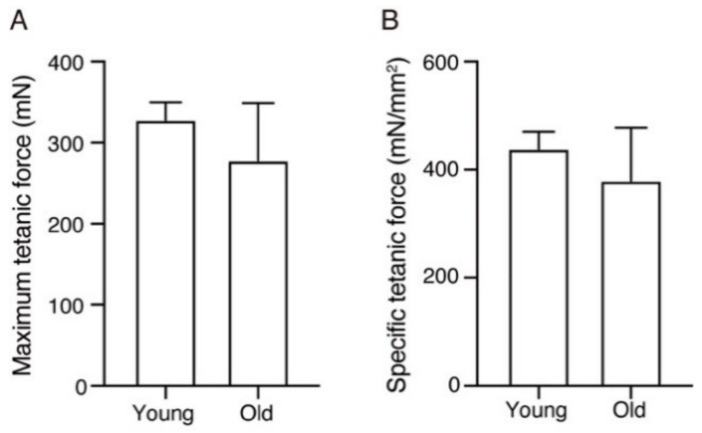
The measurement of the maximum tetanic force and specific tetanic force. (**A**) The maximum tetanic force is shown. (**B**) The specific tetanic force was calculated by dividing the absolute maximum tetanic force by the physiological cross-sectional area. Data are expressed as means ± standard deviation. *n* = 8 mice per group.

The above procedures were conducted using young and old EDL muscles. Both the absolute values of maximum tetanic force and the specific tetanic force, which represents the maximal tetanic force normalized to the muscle cross-sectional area, tended to decrease with aging although there were no statistically significant differences because of the high variability within the old group (Figure 2A,B).

**Figure 3:**
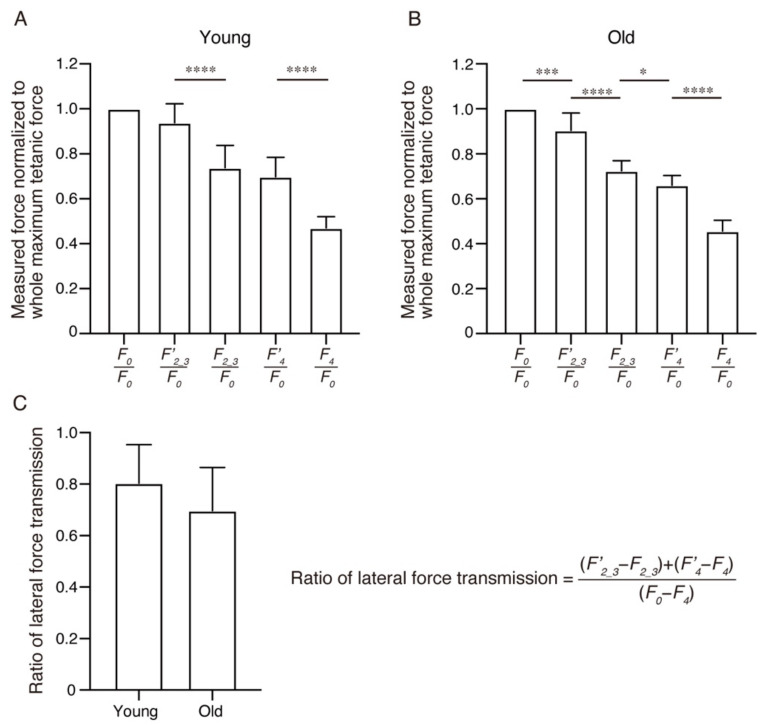
The measured force during a series of tenotomy and myotomy and total contributing ratio of lateral force transmission. (**A**) The force generated in each step in young mice was measured. The measured force was normalized to the maximum tetanic force of the whole extensor digitorum longus (EDL) muscle (*F_0_*). (**B**) The force generated in each step in old mice was measured. The measured force was normalized to the maximum tetanic force of the whole EDL muscle (*F_0_*). (**C**) The contributing ratio of lateral force transmission in total force transmission was calculated. The formula for the ratio of lateral force transmission is shown on the right. Data are expressed as means ± standard deviation; (**A**,**B**) one-way analysis of variance followed by the Holm–Šídák test or (**C**) two-sided unpaired *t*-test. *n* = 8 mice per group. * *p* < 0.05, *** *p* < 0.001, **** *p* < 0.0001.

The force measured in each step was normalized to the maximum tetanic force generated by the whole EDL muscle (*F_0_*) (Figure 3A,B). Significant differences were observed between *F′_2_3_*/*F_0_* and *F_2_3_*/*F_0_* as well as *F′_4_*/*F_0_* and *F_4_*/*F_0_* in both young and old groups, indicating that a significant portion of the force is transmitted laterally in both young and old muscles (Figure 3A,B). There were significant differences between *F_0_*/*F_0_* and *F′_2_3_*/*F_0_* as well as *F_2_3_*/*F_0_* and *F′_4_*/*F_0_* only in the old group but not in the young group, suggesting an increased dependence on longitudinal force transmission in old muscle (Figure 3A,B). Next, the total contribution ratio of the lateral force transmission was calculated, as shown in Figure 3C. The contribution ratio of lateral force transmission in total force transmission was approximately 80% in the young muscle (Figure 3C). This value is consistent with those of previous studies [3,4]. The contributing ratio of lateral force transmission tended to be low in the old muscle, although the difference was not statistically significant because of high variability (Figure 3C).

**Figure 4:**
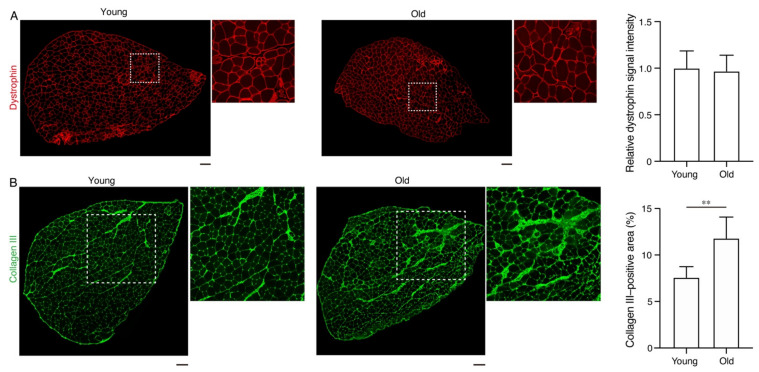
Dystrophin expression and connective tissue volume in the extensor digitorum longus (EDL) muscle. (**A**) EDL muscle sections of young and old mice were stained with a dystrophin antibody. The right panels are magnified views of boxed regions in the left panels. Relative dystrophin signal intensity is shown. (**B**) EDL muscle sections of young and old mice were stained with an antibody against collagen III. The right panels are magnified views of boxed regions in the left panels. The percentage of the collagen III-positive area is shown. Data are expressed as means ± standard deviation; the two-sided unpaired *t*-test. *n* = 5 mice per group. ** *p* < 0.01. Scale bars: 100 μm.

Since dystrophin plays a critical role in linking the myofiber cytoskeleton to the extracellular matrix [3], this molecule is thought to be involved in lateral force transmission. In both young and old muscles, all myofibers were clearly stained with a dystrophin antibody (Figure 4A). Dystrophin signal intensities were comparable between young and old groups (Figure 4A). These results indicate that the expression levels and localization of dystrophin are not affected by aging. The volume of connective tissue was subsequently examined as a mathematical model predicted that an increase in the connective tissue volume results in a decrease in the efficiency of lateral force transmission [2]. Collagen III immunostaining demonstrated that the connective tissue volume was significantly increased in the old muscle compared to that in the young muscle (Figure 4B). Thus, the increased connective tissue volume may be involved in the alteration of the force transmission property of muscles caused by aging.

## 3. Discussion

Transmitting force from the muscle to the tendon is a very important step in generating muscle strength. Several lines of evidence clearly indicate that lateral force transmission is the predominant pathway in the force transmission system of muscles. However, it is still challenging to measure lateral force transmission in mice. In this study, the lateral force transmission of the mouse EDL muscle was measured by applying a series of tenotomy and myotomy steps, a technique used in rat muscles [4]. The tenotomy and myotomy of heads II and III were modified by handling heads II and III as one head, as follows. The tenotomy of heads II and III was performed simultaneously, and myotomy between heads II and III was skipped. This study demonstrated that approximately 80% of force is transmitted through the lateral pathway in young muscles, similar to the results of previous studies [3,4]. Thus, the method used in this study enables the measurement of lateral force transmission in mouse muscles.

Although the mechanisms of age-related decrease in muscle strength remain largely unknown, non-myofiber components in skeletal muscle tissues seem to be involved in the loss of muscle strength [8,9]. Among non-myofiber components in the muscle tissue, the connective tissue may have a significant impact on the decline in muscle strength. The connective tissue plays a central role in lateral force transmission and is therefore a critical determinant of muscle strength. Aging is known to adversely affect connective tissue properties by eliciting pathological changes in the connective tissue, such as fibrosis and fat infiltration [10,11,12]. Thus, age-related changes in the connective tissue may cause a decrease in the efficiency of lateral force transmission, leading to the loss of muscle strength.

The dystrophin–glycoprotein complex (DGC) is an important component that provides a mechanical linkage between the contractile components of myofibers and the interstitial connective tissue. Therefore, the DGC is thought to be indispensable for lateral force transmission [13]. Ramaswamy et al. showed a decreased expression of dystrophin in very old rats and proposed that the disruption of the DGC is implicated in the mechanisms that underlie impaired lateral force transmission by aging [3]. In contrast, Rice et al. reported that the amount of dystrophin, β-dystroglycan, and α-sarcoglycan increased with aging in the rat EDL muscle [14]. In this study, there was no observed difference in the levels of dystrophin expression between young and old mice. The reason for the discrepancy in these studies is not clear; however, these inconsistent results suggest that the reduced levels of dystrophin cannot be a reason for the age-related change in lateral force transmission. Other factors that influence the efficiency of lateral force transmission include the thickness of the connective tissue [2]. In agreement with previous studies [3,4], a significant increase was observed in the connective tissue volume in old muscles, raising the possibility that the increased thickness of the connective tissue is one of the causal factors of age-related changes in lateral force transmission, although other factors, such as the stiffness of the connective tissue may also be involved. Molecular mechanisms by which force transmission property of muscle is regulated would be a future task. Many genetically-engineered mouse models can be useful tools to undertake this task. Therefore, the method described here will provide valuable information for such studies.

Collectively, this study evaluated the efficiency of lateral force transmission using the mouse EDL muscle and demonstrated that measurement of lateral force transmission in mice is possible. The method presented here could be used to investigate the function of the connective tissue in relation to muscle strength.

## 4. Materials and Methods

### 4.1. Mice

Thirteen-week-old and 28-month-old male C57BL/6 mice were used as young and old mice, respectively.

### 4.2. Measurement of In Vitro Contractile Force

The EDL muscles were isolated and subjected to contractile force measurements. The isolated EDL muscle was secured using a 5–0 silk suture at the distal and proximal tendons. The muscles were set in an experimental chamber filled with mammalian Ringer solution containing (mM): NaCl (137), NaHCO_3_ (24), glucose (11), KCl (5), CaCl_2_ (2), MgSO_4_ (1), NaH_2_PO_4_ (1), and tubocurarine chloride (0.025), adjusted to pH 7.4. The chamber was perfused continuously with 95% O_2_ and 5% CO_2_ and maintained at a temperature of 23 °C. The MyoDynamics Muscle Strip System (DMT, Hinnerup, Denmark, model 840MD), a square pulse electrical stimulator (DMT, model CS200); and PowerLab 4/26 data acquisition system (ADInstruments, Dunedin, New Zealand) were used to measure the contractile force. Measurements were performed according to a previously described method [15]. Square wave pulses 1.0 ms in duration were generated by a stimulator and the maximal stimulus was determined by adjusting the current to obtain the maximal twitch tension, and subsequently, the stimulus at 20% above the maximal was set to achieve the supramaximal stimulus. Optimal muscle length was determined by gradually stretching the muscle until there was no further increase in the twitch tension. A train of supramaximal stimuli for 300 ms at 150 Hz at the optimal length was applied to measure the maximal tetanic force. Subsequently, a series of tenotomy and myotomy procedures were conducted according to a previous study [4] with minor modifications. After the measurement of the maximal tetanic force, the muscle was removed from the chamber and transferred to a dish containing mammalian ringer solution. Under a stereoscopic microscope (Zeiss, Jena, Germany), the tendons of heads II and III were cut, and the muscle was set again in the chamber. Thereafter, the same electrical stimulation was applied to the muscle, and the tetanic force was recorded. The muscle was removed from the chamber and transferred to a dish. Heads II and III were separated from head IV under a stereoscopic microscope, and the muscle was set again in the chamber. The same electrical stimulation was applied to the muscle, and the tetanic force was recorded. Similar tenotomy and myotomy procedures were applied to head IV, and the force measured after each step was recorded. After the analysis of force generation, muscle length was measured, and then the muscles were removed from the chamber, blotted to dry, and weighed. The absolute force was normalized with the physiological cross-sectional area, which was computed as the product of the ratio of the muscle weight and length and the density for mammalian skeletal muscle, 1.066 mg/mm^3^, to obtain the specific force. Both right and left EDL muscles were subjected to analysis and the force was determined by calculating mean value.

### 4.3. Immunofluorescent Staining and Microscopy

Fresh muscle samples were rapidly frozen in isopentane cooled with liquid nitrogen. Different mice from those used for the force measurement were used for immunohistological staining. Using a cryostat, 7 μm-thick frozen sections were prepared. Fresh frozen sections were fixed with acetone for 5 min at −20 °C and blocked with a protein block serum-free reagent (Agilent, Santa Clara, CA, USA) for 15 min. The specimens were incubated with primary antibodies at 4 °C overnight, followed by secondary staining. The primary and secondary antibodies used were anti-dystrophin (1:800; Abcam, Cambridge, UK, Cat# ab15277), anti-collagen III (1:100; Abcam, #ab7778), Alexa Fluor 488 anti-rabbit IgG (1:1000; Jackson ImmunoResearch, West Grove, PA, USA, #711-545-152), and Alexa Fluor 594 anti-rabbit IgG (1:1000; Jackson ImmunoResearch, #711-585-152). Stained samples were mounted with SlowFade Diamond anti-fade reagent (Thermo Fisher, Waltham, MA, USA, S36972). Immunofluorescent images were obtained using an inverted fluorescence microscope DMI6000B (Leica, Wetzlar, Germany), BZ-X710 (Keyence, Osaka, Japan), and a confocal laser scanning microscope system TCS SP8 (Leica). Quantitative analyses of dystrophin and collagen III staining were performed using the Hybrid Cell Count application (Keyence).

### 4.4. Statistical Analysis

All quantitative analyses were performed in a blinded manner. Statistical significance was assessed using GraphPad Prism 8 (ver. 8.4.1, GraphPad Software, San Diego, CA, USA). Two-tailed unpaired student’s *t*-tests were used for comparisons between the two groups. For comparisons among more than two groups, one-way analysis of variance followed by the Holm–Šídák test was used. Statistical significance was set at *p* < 0.05.
